# Deep Convolution Neural Network for Laryngeal Cancer Classification on Contact Endoscopy-Narrow Band Imaging

**DOI:** 10.3390/s21238157

**Published:** 2021-12-06

**Authors:** Nazila Esmaeili, Esam Sharaf, Elmer Jeto Gomes Ataide, Alfredo Illanes, Axel Boese, Nikolaos Davaris, Christoph Arens, Nassir Navab, Michael Friebe

**Affiliations:** 1 INKA—Innovation Laboratory for Image Guided Therapy, Otto-von-Guericke University Magdeburg, 39120 Magdeburg, Germany; esam.sharaf@ovgu.de (E.S.); elmer.gomesataide@ovgu.de (E.J.G.A.); alfredo.illanes@med.ovgu.de (A.I.); axel.boese@med.ovgu.de (A.B.); michael.friebe@ovgu.de (M.F.); 2 Chair for Computer Aided Medical Procedures and Augmented Reality, Technical University of Munich, 85748 Munich, Germany; nassir.navab@tum.de; 3 Department of Nuclear Medicine, Medical Faculty, Otto-von-Guericke University Magdeburg, 39120 Magdeburg, Germany; 4 Department of Otorhinolaryngology, Head and Neck Surgery, Magdeburg University Hospital, 39120 Magdeburg, Germany; nikolaos.davaris@med.ovgu.de; 5 Department of Otorhinolaryngology, Head and Neck Surgery, Giessen University Hospital, 35392 Giessen, Germany; christoph.arens@hno.med.uni-giessen.de; 6 IDTM GmbH, 45657 Recklinghausen, Germany

**Keywords:** Deep Convolution Neural Network, contact endoscopy, narrow band imaging, classification, larynx, cancer

## Abstract

(1) Background: Contact Endoscopy (CE) and Narrow Band Imaging (NBI) are optical imaging modalities that can provide enhanced and magnified visualization of the superficial vascular networks in the laryngeal mucosa. The similarity of vascular structures between benign and malignant lesions causes a challenge in the visual assessment of CE-NBI images. The main objective of this study is to use Deep Convolutional Neural Networks (DCNN) for the automatic classification of CE-NBI images into benign and malignant groups with minimal human intervention. (2) Methods: A pretrained Res-Net50 model combined with the cut-off-layer technique was selected as the DCNN architecture. A dataset of 8181 CE-NBI images was used during the fine-tuning process in three experiments where several models were generated and validated. The accuracy, sensitivity, and specificity were calculated as the performance metrics in each validation and testing scenario. (3) Results: Out of a total of 72 trained and tested models in all experiments, Model 5 showed high performance. This model is considerably smaller than the full ResNet50 architecture and achieved the testing accuracy of 0.835 on the unseen data during the last experiment. (4) Conclusion: The proposed fine-tuned ResNet50 model showed a high performance to classify CE-NBI images into the benign and malignant groups and has the potential to be part of an assisted system for automatic laryngeal cancer detection.

## 1. Introduction

Laryngeal cancer is one of the most common malignancies in the head and neck area, with a growing incidence rate every year [1]. The treatment options and prognosis depend on the cancer stage at the time of diagnosis. Precancer or early-stage laryngeal cancer is associated with high rates of laryngeal preservation, a local control rate of 87–89%, and a favorable prognosis [2]. On the other hand, advanced-stage cancer requires multi-modal treatment strategies resulting in significant toxicities and a poorer quality of life. Despite optimized treatment schemes, studies report high recurrence rates and a 5 year overall survival of 33–61% [3,4].

Nowadays, the endoscopic imaging modalities have become the standard procedure for screening and early diagnosis of laryngeal cancerous and precancerous lesions in clinical settings. These methods are widely applicable before performing a surgical biopsy for histological tissue examination in the context of the so-called optical biopsy [5,6]. As one of these techniques, the combination of Contact Endoscopy (CE) with Narrow Band Imaging (NBI) can represent an enhanced and magnified visualization of changes in the morphology and three-dimensional orientation of vocal fold’s subepithelial blood vessels [7,8]. The visual evaluation of these vascular structures in CE-NBI images can provide complementary information for the diagnosis of laryngeal cancerous or precancerous lesions. However, the use of CE-NBI for diagnosis highly relies on the experience of the otolaryngologists and requires several years of training. This can result in a subjective decision process followed by an overtreatment or undertreatment planning [7,9,10].

The advanced development of feature engineering, Machine Learning (ML), and Deep Learning (DL) methods in the area of medical applications provides several paths to assist the clinicians and overcome such challenges in the clinical environments. In this regard, several computer-based approaches were used on the larynx endoscopic images. These methods can assist otolaryngologists by providing complementary information regarding the stage of the cancer and characteristics of the vascular trees and larynx epithelial tissue [11]. In the area of laryngoscopic and NBI image analysis, an ensemble of Convolutional Neural Networks (CNN) with texture and frequency-domain-based features [12] and a set of hand-crafted texture and first-order statistical features [13] were proposed for larynx cancerous tissue classification. A Deep Convolutional Neural Network (DCNN) achieved the overall accuracy of 86% to detect cancer, precancerous lesions, and normal tissues in larynx [14]. A image classification system based on CNN outperformed the manual assessment of trainees in discriminating cysts, granulomas, nodules, normal cases, palsies, papillomas, and polyps [15]. The combination of hand-crafted and DL-based features showed a median classification recall of 98% for the diagnosis of early stage Squamous Cell Carcinoma (SCC) in larynx [16]. Moreover, another CNN-based approach achieved an equivalent performance to otolaryngologists’ predictions for the diagnosis of laryngeal SCC [17].

Given that there is a need for more magnified and enhanced endoscopic techniques such as CE-NBI images, two sets of hand-crafted features combined with ML techniques were proposed for the automatic assessment of these type of images. These methods have the potential to provide an evaluation of vascular characteristics [18,19], assist otolaryngologists when there are disagreements regarding the final diagnosis [20,21], and present a computer-based classification of benign and malignant laryngeal lesions [22]. However, these works exhibited certain drawback in terms of the multiple image preprocessing stages that resulted in the loss of information from the images as well as manual feature extraction processes. Additionally, these studies focused only on some specific characteristics of the CE-NBI images, such as vascular geometry and textural characteristics and not the structures as a whole.

The main objective of this study is to use a fully automatic CE-NBI endoscopic image-based DCNN approach for the classification of laryngeal lesions and provide an objective assessment for otolaryngologists during the treatment process. This is performed to circumvent the disadvantages posed by ML-based approaches and rather have an approach that is more streamlined and automatic with minimal human intervention in the classification of lesions. To our knowledge, this is the first study that applies a DCNN-based approach for larynx CE-NBI image classification. The proposed approach uses the transfer learning concept which includes a pretrained ResNet50 model instead of developing a network from the scratch. Moreover, the pretrained ResNet50 model was tuned and combined with cut-off-layer technique to achieve the optimum architecture for this classification task. The performance of the proposed approach was evaluated in three different experiments. Then, it was compared to the performance of the state-of-the-art methods in the area of CE-NBI image classification.

## 2. Materials and Methods

In this section, we highlight the aspects of data preparation, discuss the model architecture, and detail the steps carried out during the experiments and training of the DCNN.

### 2.1. Data Preparation

CE-NBI video scenes of 146 patients who went through a microlaryngoscopy procedure were captured using an Evis Exera III Video System with integrated NBI-filter (Olympus Medical Systems, Hamburg, Germany). This setup included a rigid 30-degree contact endoscope (Karl Storz, Tuttlingen, Germany) with a fixed magnification of 60×. Then, 8181 CE-NBI images were extracted from the videos as explained in Esmaeili et al. [7,18]. We went through each video scene and manually selected the time intervals where the video quality was good enough to visualize the blood vessels. Then, one in every ten frames was automatically extracted from the selected intervals in JPEG format images (1008×1280 pixels) to have unique and nonredundant vascular pattern in CE-NBI images. All patients’ data were pseudonymized, and only biopsy results were taken to label images into benign and malignant lesions according to the WHO classification [23]. The benign class has 5313 images of patients with histopathologies such as Cyst, Polyp, Reinke’s edema, Papillomatosis, Hyperplasia, Hyperkeratosis, and Mild Dysplasia. The malignant group includes 2868 images of patients diagnosed with Moderate Dysplasia, Severe Dysplasia, and Carcinoma in situ and SCC. The data were preprocessed and prepped in terms of size before being used as an input for the DCNN.

### 2.2. Model Architecture

The DCNN architecture used in this study is discussed here. DCNNs have gained recognition due to their adaptability for image recognition problem statements. These networks also yield higher accuracies as compared to other ML methods, due to their ability to solve problems from end-to-end rather than breaking them down as in the case of ML.

**Figure 1:**
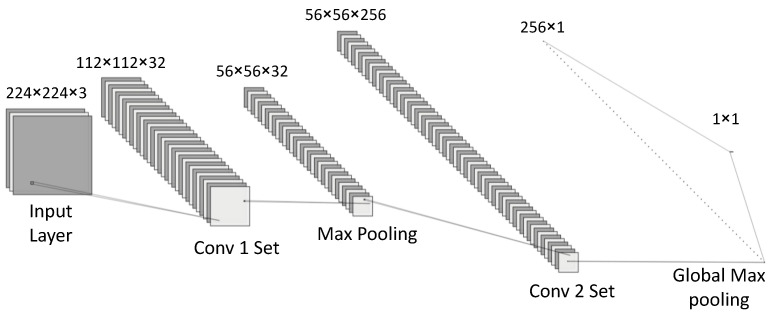
The overall architecture of the proposed approach.

Transfer learning concept has become an important part of the growth of DL-based approaches in the field of medical image classification. It provides the chance of reusing a pretrained model as a starting point for a new classification task with comparatively few data. The pretrained network is a network that has already been introduced to a specific dataset and learned to extract valuable features from it. The dataset used for the pretraining is not always the same as the actual dataset for the second classification task, but the extracted features are similar in nature. This network can then be used as a starting point to learn a new classification task. In this study, a pretrained ResNet50 on ImageNet [24] database was considered for CE-NBI image classification task. Residual Networks (ResNets) are considered as examples of very deep classic structures in the computer vision literature [25]. ResNet50 is 50 layers deep, and the deepness level is related to the network’s capability to capture high (or higher) patterns. ResNets optimize toward zero, which in turn accelerates the convergence to the optimal point in the solution space, instead of a real number. Batch normalization is another interesting feature that is embedded in ResNet’s structure. It speeds up the convergence and in doing so reduces the training epochs required. It also has a regularization effect during the training phase. Figure 1 shows the overall view of the proposed architecture.

The pretrained ResNet50 was combined with the fine-tuning strategy as well as cut-off-layer technique to obtain the optimum performance for CE-NBI image classification. Fine-tuning a pretrained DCNN is beneficial as it enables the user to speed up training and overcome smaller dataset sizes. The fine-tuning technique wherein all the layers were fine-tuned was adopted for this work. In order to account for the issue of overfitting of ResNet50, we proposed setting the cut-off-layer to discard part of the network. The cut-off layer is the last layer in feature extraction part of the network, where the classifier part begins. This layer tends to be where the activation occurs. While training the network, it was noted that overfitting occurred due to the large size of the original ResNet architecture. Hence, the cut-off layer was set empirically. This resulted in several models with different layer counts and therefore feature counts depending on where the cut-off layer was set. The final cut-off layer was selected based on the overall performance of the network. Then, different variations of the model were implemented for having sufficient number of features in a trade off between the training stage success and generalization ability of the model on unseen images.

### 2.3. Experiments

The experiments for this work were divided into three parts as shown in Table 1. A total of three experiments were conducted to determine the model most suitable for our problem statement. In the conducted experiments, a total of 72 models were trained and tested using the data collected. The main difference between these experiments was related to the strategy of data separation. Apart from this, a few experiments also took into consideration different network hyperparameters and changes in the volume of data. In Experiment 1, the separation into training and testing sets was performed randomly to form a 80–20 train-test split. Additionally, different cut-off-layer strategies and classifiers were tested in this experiment. In Experiment 2, we employed a manual method for splitting the training and testing data. This was performed so as to ensure that none of the test data were part of the training data as well as the images of patients exclusively tied to separate sets. Then, the best-performed model from Experiment 1 was tested in this experiment. In Experiment 3, data augmentation (vertical and horizontal flipping) was applied, and testing data selection criteria were kept the same as Experiment 2. The best-performed model from Experiment 1 was also tested under the specified condition of Experiment 3.

### 2.4. Training Details

The ResNet50 model was adopted as the backbone for this work. Input images were resized to 224×224 pixels in the preprocessing stage. Data augmentation on the images was performed by employing the horizontal and vertical flipping methods. Binary cross entropy was used as a loss function along with Stochastic Gradient Descent (SGD) as the optimizer. The parameters were tuned as follows: batch_size = 32, learning_rate = 0.001, decay = $1\times 10^{-6}$, momentum = 0.9, Nesterov momentum = True. The cut-off layer was set at “conv2_block3_out” in an iterative process. Early stoppage was also set with a patience of 5 epochs. The network was trained for a total of 35 epochs and programmed using Python version 3.8.8. The study was carried out on a deep learning workstation with and Nvidia Quadro P6000 GPU. The 5-fold crossvalidation technique was used for validating the models.

### 2.5. Performance Metrics

The study used accuracy, sensitivity, and specificity as performance metrics. These are given below along with their formulas:

(1) $$Accuracy=\frac{TruePositives+TrueNegatives}{TotalNumberofImages}$$

(2) $$Sensitivity=\frac{TruePositives}{TruePositives+FalseNegatives}$$

(3) $$Specificity=\frac{TrueNegatives}{TrueNegatives+FalsePositives}$$

## 3. Results

The performance of the selected models from the three experiments are listed in Table 2. On average, 69.9 min was taken to execute the training and validation phase during different experiments, followed by a testing phase that took on average 52.3 s.

**Figure 2:**
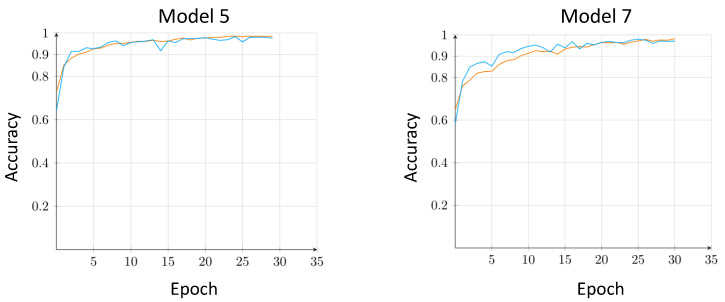
Comparison of the accuracy track between Model 5 and Model 7 in Experiment 1. Orange and blue lines represent the training and validation phase, respectively.

Of the all models trained and tested, Models 5–7 showed the most promising results during Experiment 1. Model 5 achieved an accuracy, sensitivity, and specificity of 0.979, 0.967, and 0.986, respectively. When compared to the metrics produced by Model 6 and Model 7, these scores were higher in both the validation and testing phases. Figure 2 shows the comparison between the accuracy curves between Models 5 and 7 over 35 epochs for Experiment 1. It can be seen from the figure that the curves for Model 5 are more consistent as opposed to the curves seen in Model 7 in the this experiment. On the other hand, by visual evaluation of the graph, we can see that the accuracy achieved by Model 5 at epoch 5 is equal to 0.927, while Model 7 had a lower rate equal to 0.853% at the same epoch. Based on these evaluations, we decided to move forward with Model 5 and Global Max Pooling classifier for the following two experiments.

In Experiment 2, Model 5 exhibited marginally lower scores in terms of validation accuracy, sensitivity, and specificity where the testing data were manually selected so as to ensure they were not part of the training set. In this experiment, the deviation in accuracy value occurs between validation and testing scenarios because there is the possibility that the validation set is not representative to the testing dataset. This can lead to biased fine-tuned model to the validation set and possible overfitting in this scenario. Therefore, we moved on to Experiment 3 with Model 5 and Global Max Pooling classifier together with data augmentation techniques.

**Figure 3:**
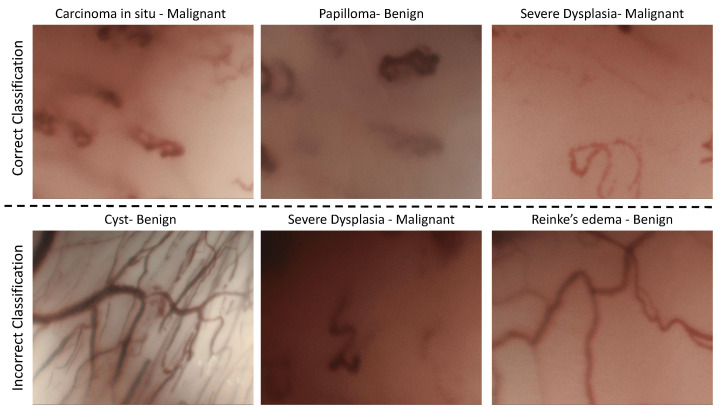
The example of correct and incorrect classification of CE-NBI images in Experiment 3.

Model 5 in Experiment 3 exhibited an accuracy, sensitivity, and specificity of 0.925, 0.888, and 0.960, respectively, during the validation phase and an accuracy score of 0.835 in the testing scenario. Figure 3 depicts the examples of the classification given by Model 5. The top row of the Figure 3 corresponds to accurately classified images and the bottom row to inaccurately image classifications. The Perpendicular Vascular Changes (PVC) in laryngeal Papillomatosis can be difficult to visually distinguish from PVC in premalignant and malignant histopathologies [26]. Among the accurate classifications represented in Figure 3, it is significant to note that Model 5 was able to accurately differentiate such images where there were similar vascular structures but different histopathologies (malignant Carcinoma in situ vs. benign Papilloma). On the other hand, classification inaccuracies can arise due to the complexity of the vessel arrangements in the CE-NBI images. This issue was predicted in Experiment 3 as the testing data included a set of unseen and augmented images. Moreover, the dataset has a comprehensive selection of several histopathologies from different patients that can increase the chance of complexity during classification scenarios of the unseen and augmented data.

**Figure 4:**
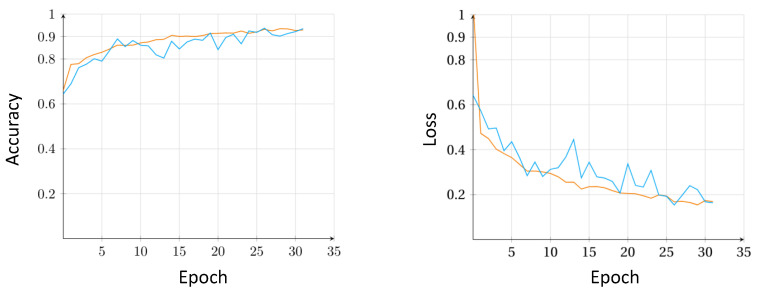
The accuracy and loss graphs of model Model 5 in Experiment 3. Orange and blue lines represent the training and validation phase, respectively.

Figure 4 depicts the graphs of the accuracy and loss for Model 5 in Experiment 3. Both graphs follow a smooth ascend (accuracy) and descend (loss). From this, we can infer that the model followed a relatively stable training cycles through each of the epochs. The accuracy (training vs. validation) graph show a good fit overall for the model during the experiment. Although they meet in the end, the loss (training vs. validation) graph shows a much more erratic behavior during the epochs.

**Figure 5:**
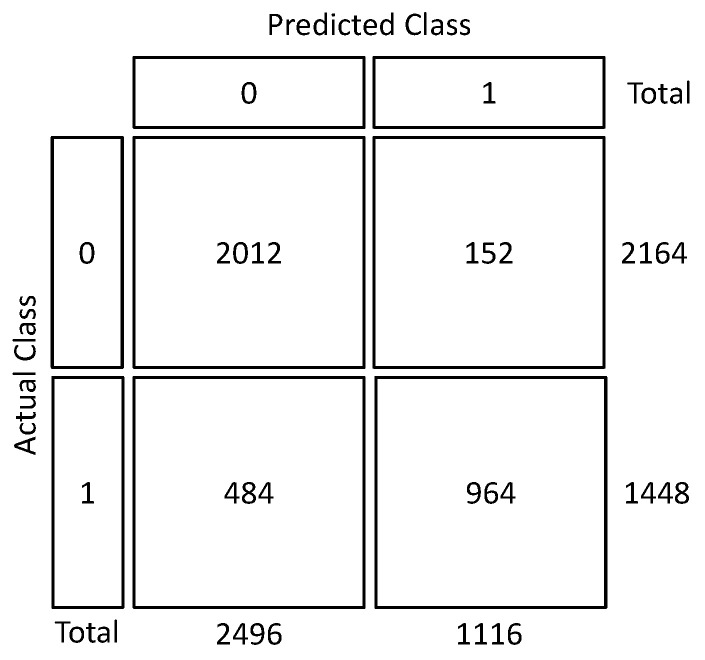
Confusion matrix testing scenario of Experiment 3.

Figure 5 exhibits the confusion matrix of Model 5 in testing scenario of Experiment 3. The images in the benign and malignant groups were labeled as 0 and 1, respectively. With this explanation, it can be seen from this matrix that the number of misclassified images in the malignant group is more than the benign class.

## 4. Discussion

In this study, a fully automatic DCNN-based approach using a pretrained and fine-tuned ResNet50 architecture was adopted and evaluated on CE-NBI images for the benign and malignant laryngeal lesion classification. To the best of our knowledge, no previous study has applied DCNN-based models on larynx CE-NBI images for any classification or segmentation purposes. Considering the presented results, the DCCN-based approach has the potential to differentiate malignant lesions from several benign ones in CE-NBI images with high performance and can provide a more consistent interpretation and an objective decision-making process for clinicians.

The application of DCNN-based methods has brought effective solutions in the area of image analysis for a better understanding of image content. Together with the development of these techniques, the concept of transfer learning has introduced a new perception to deal with the problem of a limited number of images for training these models. It allows reusing the pretrained models for a similar task, such as image classification. Among DCNNs that achieved significant outcomes, AlexNet [27], VGGNets [28], InceptionNets [29], and ResNets [25] are some well-known pretrained models for medical image classification. These architectures were developed for certain purposes and have shown their own strengths and limitations. Depending on the area of application as well as the type of imaging modality, each of these networks has shown the ability to provide a better understanding of the patients’ status for the clinicians [30,31,32]. Among them, the ResNet convolutional networks are the most popular as they can offer very deep architectures with shortcut connections to solve the vanishing gradient problem. Moreover, the batch normalization features in these networks can speed up the convergence and reduce the required training epochs [25]. In the area of medical image analysis, ResNet34 was evaluated to determine the class of laryngeal Stimulated Raman Scattering (SRS) images based on normal or neoplastic classes. This architecture showed the rapid and automated recognition on the validation set with an accuracy of 0.959 [33]. In another study, a fine-tuned ResNet50 network was used for classifying multimodal images of breast tissues into normal, fat, and cancerous. Using leave-one-patient-out crossvalidation, the model achieved the mean sensitivity of 0.862 on the validation images [34]. In addition, fine-tuned ResNet50, InceptionV2, and SqueezeNet models were selected to multiclassify laryngoscopy frames into four classes and were achieved the macroaverage AUC (Area Under the Curve) of 0.998, 0.989, and 0.999, respectively [35]. In a recent evaluation, ResNet50 and ResNet101 architectures were part of an ensemble model that was applied for cancer tissue classification in larynx NBI images. The combination of this ensemble model with a series of hand-crafted features achieved the classification accuracy of 0.954 [12]. Considering the proven performance of ResNet convolutional networks in medical image classification tasks as well as the advantages of these architectures over other networks, the pretrained ResNet50 was used for our evaluation. This network utilized images in the pretraining step that displayed a pattern similar to that of the blood vessels as used in this study.

After the evaluation, the outcomes of three different experiments, the fine-tuned ResNet50 model from the Experiment 3 was proposed as the final architecture from 72 total models. This model achieved the mean accuracy, sensitivity, and specificity of 0.925, 0.888, and 0.960 in the validation phase and the mean accuracy of 0.835 from the testing scenario. Although this model showed lower performance than the tested models in Experiments 1 and 2, it was evaluated in a more realistic scenario. One of the major benefits of this model over the latest DCNN-based methods is the size of the fine-tuned ResNet50 model. The application of the cut-of-layer technique resulted in a smaller model that only has the size equal to ≈1% of the full ResNet50 architecture (1.96 Megabytes versus 180.65 Megabytes). In addition, the smaller architecture showed faster training with less prone to result in overfitting. Earlier, it was mentioned that the chance of overfitting increases while using the ResNet50 architecture. Hence, apart from cut-off-layer technique, other strategies such as including a larger number of images, performing data augmentation, and early stopping were also employed to avoid the overfitting of ResNet50 in this study.

In comparison to the other works in the area of laryngeal cancer detection and classification, we used the CE-NBI images as the imaging modality. NBI imaging enables a highly contrasted visualization of vascular structures. The essential advantage of CE-NBI over the normal white light laryngoscopy is the highly magnified visualization of vascular patterns that results in a more precise evaluation of laryngeal lesions [7].

In this study, there is a slight data imbalance between the number of benign and malignant images in the CE-NBI image dataset (≈60% benign vs. ≈40% malignant). This issue could be solved by using a two-fold data augmentation approach where the data augmentation is first performed to balance the data and then the second augmentation is applied to the entire dataset as a whole. However, this can increase the risk of redundancies especially in the case of CE-NBI images as vascular patterns are already very similar. For this reason, we chose not to tamper with the imbalance issue because it is not significantly greater than it would affect the performance of the network. Moreover, the data, as they are, are representative of the true clinical scenario where there is often an imbalance in the data collected. This dataset includes around 8000 CE-NBI images from a wide range of various histopathologies in both benign and malignant groups, which is a comparable number of data in comparison to other studies where the endoscopy-based imaging techniques were used for similar classification tasks in the larynx. The number of images on these evaluations ranges from a minimum of 330 to a maximum of 14,000 [12,13,14,15,16,35]. This maximum number exists because multiple clinical centers were in the data collection process simultaneously [14]. On the other hand, the subsets of this CE-NBI image dataset were used to develop and test multiple hand-crafted feature extraction and ML methods for laryngeal cancer classification [18,20,22]. In this respect, the recent work reported the classification accuracy of 0.966 using two feature sets combined with k-Nearest Neighbors (kNN) classifier [22]. Even though this method outperformed the proposed model, it included three different image preprocessing stages, needed the manual parameter selections, and was tested on a smaller dataset.

As was mentioned before, the benign lesions show similar vascular patterns to the malignant ones in CE-NBI image analysis. The visual evaluation of this cases can cause one of the serious problems in the clinical environment which is the differentiation between benign and malignant lesions [20]. In the present study, the achieved specificity was higher than the sensitivity values in all experiments. This outcome can emphasize the ability of the proposed model to overcome this issue and assist otolaryngologists to also evaluate benign cases more confidently.

## 5. Conclusions

In summary, a CE-NBI endoscopic image-based DCNN model was developed and tested through a fine-tuned ResNet50 architecture. The proposed model had a high performance for the automatic classification of laryngeal cancerous lesions and showed comparable performance to the studies in the area of larynx CE-NBI image classification, as was explained in the previous section. The proposed structure is significantly smaller than the full ResNet50 architecture as a result of the cut-off-layer technique. Moreover, no over- and under-fitting were observed in the final architecture. The proposed model has the potential to be a solution for the subjective assessment of the benign and malignant laryngeal lesions in clinical settings and reduce the chance of performing an invasive surgical biopsy. This effective solution can be part of the Compute-Aided-Diagnosis (CAD) system that assists otolaryngologists during the decision-making process and improves the optical diagnosis rate of larynx cancer.

To improve the performance of the proposed model, more investigations are planned for multidomain feature extraction methods (DCNN combined with hand-crafted features) as well as the development of ensemble DCNN models for the future work. Moreover, it is essential to continue further development on a multiclassification scenarios to differentiate between different laryngeal histopathologies and improve the application of optical biopsy in the clinical settings.

## Figures and Tables

**Table 1:** The summary of three experiments classified according to the different conditions.

Experiment	Data Augmentation	Cut-Off Layer	Classifier	Dataset Separation
Experiment 1	No	conv2_block3_out (230 K parameters)	Global Max Pooling	Random
		conv2_block3_out (230 K parameters)	Global Max Pooling + Dropout	
		No cut-off (23.5 M parameters)	Global Max Pooling	
Experiment 2	No	conv2_block3_out (230 K parameters)	Global Max Pooling	Manual
Experiment 3	Yes	conv2_block3_out (230 K parameters)	Global Max Pooling	Manual

**Table 2:** Results of the selected models in each experiment. Metrics of the validation and testing phases are averages over five folds.

Experiment	Model	Validation				Testing
		Accuracy	Sensitivity	Specificity	Loss	Accuracy
Experiment 1	Model 5	0.979	0.967	0.986	0.06	0.991
	Model 6	0.943	0.914	0.959	0.15	0.958
	Model 7	0.967	0.960	0.974	0.11	0.984
Experiment 2	Model 5	0.976	0.958	0.985	0.07	0.929
Experiment 3	Model 5	0.925	0.888	0.960	0.20	0.835

## Data Availability

The data that support the findings of this study are part of the research project and are not publicly available.

## References

[1] Wang J.Y., Zhang Q.W., Wen K., Wang C., Ji X., Zhang L. (2021). Temporal trends in incidence and mortality rates of laryngeal cancer at the global, regional and national levels, 1990–2017. BMJ Open.

[2] Guimarães A.V., Dedivitis R.A., Matos L.L., Aires F.T., Cernea C.R. (2018). Comparison between transoral laser surgery and radiotherapy in the treatment of early glottic cancer: A systematic review and meta-analysis. Sci. Rep..

[3] García-León F.J., García-Estepa R., Romero-Tabares A., Borrachina J.G.M. (2017). Treatment of advanced laryngeal cancer and quality of life. Systematic review. Acta Otorrinolaringol..

[4] Elicin O., Giger R. (2020). Comparison of current surgical and non-surgical treatment strategies for early and locally advanced stage glottic laryngeal cancer and their outcome. Cancers.

[5] Missale F., Taboni S., Carobbio A.L.C., Mazzola F., Berretti G., Iandelli A., Fragale M., Mora F., Paderno A., Del Bon F. (2021). Validation of the European Laryngological Society classification of glottic vascular changes as seen by narrow band imaging in the optical biopsy setting. Eur. Arch. Oto-Rhino-Laryngol..

[6] Lauwerends L.J., Galema H.A., Hardillo J.A., Sewnaik A., Monserez D., van Driel P.B., Verhoef C., Baatenburg de Jong R.J., Hilling D.E., Keereweer S. (2021). Current Intraoperative Imaging Techniques to Improve Surgical Resection of Laryngeal Cancer: A Systematic Review. Cancers.

[7] Davaris N., Lux A., Esmaeili N., Illanes A., Boese A., Friebe M., Arens C. (2020). Evaluation of Vascular Patterns using Contact Endoscopy and Barrow-Band Imaging (CE-NBI) for the Diagnosis of Vocal Fold Malignancy. Cancers.

[8] Puxeddu R., Sionis S., Gerosa C., Carta F. (2015). Enhanced contact endoscopy for the detection of neoangiogenesis in tumors of the larynx and hypopharynx. Laryngoscope.

[9] Mannelli G., Cecconi L., Gallo O. (2016). Laryngeal preneoplastic lesions and cancer: Challenging diagnosis. Qualitative literature review and meta-analysis. Crit. Rev. Oncol./Hematol..

[10] Mehlum C.S., Døssing H., Davaris N., Giers A., Grøntved Å.M., Kjaergaard T., Möller S., Godballe C., Arens C. (2020). Interrater variation of vascular classifications used in enhanced laryngeal contact endoscopy. Eur. Arch. Oto-Rhino-Laryngol..

[11] Singh V.P., Maurya A.K. (2021). Role of Machine Learning and Texture Features for the Diagnosis of Laryngeal Cancer. Mach. Learn. Healthc. Appl..

[12] Nannia L., Ghidoni S., Brahnam S. (2020). Ensemble of convolutional neural networks for bioimage classification. Appl. Comput. Inform..

[13] Moccia S., De Momi E., Guarnaschelli M., Savazzi M., Laborai A., Guastini L., Peretti G., Mattos L.S. (2017). Confident texture-based laryngeal tissue classification for early stage diagnosis support. J. Med. Imaging.

[14] Xiong H., Lin P., Yu J.G., Ye J., Xiao L., Tao Y., Jiang Z., Lin W., Liu M., Xu J. (2019). Computer-aided diagnosis of laryngeal cancer via deep learning based on laryngoscopic images. EBioMedicine.

[15] Cho W.K., Lee Y.J., Joo H.A., Jeong I.S., Choi Y., Nam S.Y., Kim S.Y., Choi S.H. (2021). Diagnostic Accuracies of Laryngeal Diseases Using a Convolutional Neural Network-Based Image Classification System. Laryngoscope.

[16] Araújo T., Santos C.P., De Momi E., Moccia S. (2019). Learned and handcrafted features for early-stage laryngeal SCC diagnosis. Med. Biol. Eng. Comput..

[17] Hu R., Zhong Q., Xu Z., Huang L., Cheng Y., Wang Y., He Y. (2021). Application of deep convolutional neural networks in the diagnosis of laryngeal squamous cell carcinoma based on narrow band imaging endoscopy. Chin. J. Otorhinolaryngol. Head Neck Surg..

[18] Esmaeili N., Illanes A., Boese A., Davaris N., Arens C., Friebe M. (2019). Novel Automated Vessel Pattern Characterization of Larynx Contact Endoscopic Video Images. Int. J. Comput. Assist. Radiol. Surg..

[19] Esmaeili N., Illanes A., Boese A., Davaris N., Arens C., Friebe M. A Preliminary Study on Automatic Characterization and Classification of Vascular Patterns of Contact Endoscopy Images. Proceedings of the 2019 41st Annual International Conference of the IEEE Engineering in Medicine and Biology Society (EMBC).

[20] Esmaeili N., Illanes A., Boese A., Davaris N., Arens C., Navab N., Friebe M. (2020). Laryngeal Lesion Classification based on Vascular Patterns in Contact Endoscopy and Narrow Band Imaging: Manual versus Automatic Approach. Sensors.

[21] Esmaeili N., Illanes A., Boese A., Davaris N., Arens C., Navab N., Friebe M. (2020). Manual versus Automatic Classification of Laryngeal Lesions based on Vascular Patterns in CE+NBI Images. Curr. Dir. Biomed. Eng..

[22] Esmaeili N., Boese A., Davaris N., Arens C., Navab N., Friebe M., Illanes A. (2021). Cyclist Effort Features: A Novel Technique for Image Texture Characterization Applied to Larynx Cancer Classification in Contact Endoscopy—Narrow Band Imaging. Diagnostics.

[23] Gale N., Hille J., Jordan R.C., Nadal A., Williams M.D. (2019). Regarding Laryngeal precursor lesions: Interrater and intrarater reliability of histopathological assessment. Laryngoscope.

[24] Deng J., Dong W., Socher R., Li L.J., Li K., Fei-Fei L. Imagenet: A large-scale hierarchical image database. Proceedings of the 2009 IEEE Conference on Computer Vision and Pattern Recognition.

[25] He K., Zhang X., Ren S., Sun J. Deep residual learning for image recognition. Proceedings of the IEEE Conference on Computer Vision and Pattern Recognition.

[26] Arens C., Piazza C., Andrea M., Dikkers F.G., Gi R.E.T.P., Voigt-Zimmermann S., Peretti G. (2016). Proposal for a descriptive guideline of vascular changes in lesions of the vocal folds by the committee on endoscopic laryngeal imaging of the European Laryngological Society. Eur. Arch. Oto-Rhino-Laryngol..

[27] Krizhevsky A., Sutskever I., Hinton G.E. (2012). Imagenet classification with deep convolutional neural networks. Adv. Neural Inf. Process. Syst..

[28] Simonyan K., Zisserman A. (2014). Very deep convolutional networks for large-scale image recognition. arXiv.

[29] Szegedy C., Liu W., Jia Y., Sermanet P., Reed S., Anguelov D., Erhan D., Vanhoucke V., Rabinovich A. Going deeper with convolutions. Proceedings of the IEEE Conference on Computer Vision and Pattern Recognition.

[30] Sarvamangala D., Kulkarni R.V. (2021). Convolutional neural networks in medical image understanding: A survey. Evol. Intell..

[31] Upreti M., Pandey C., Bist A.S., Rawat B., Hardini M. (2021). Convolutional Neural Networks in Medical Image Understanding. Aptisi Trans. Technopreneurship (ATT).

[32] Yadav S.S., Jadhav S.M. (2019). Deep convolutional neural network based medical image classification for disease diagnosis. J. Big Data.

[33] Zhang L., Wu Y., Zheng B., Su L., Chen Y., Ma S., Hu Q., Zou X., Yao L., Yang Y. (2019). Rapid histology of laryngeal squamous cell carcinoma with deep-learning based stimulated Raman scattering microscopy. Theranostics.

[34] Ali N., Quansah E., Köhler K., Meyer T., Schmitt M., Popp J., Niendorf A., Bocklitz T. (2019). Automatic label-free detection of breast cancer using nonlinear multimodal imaging and the convolutional neural network ResNet50. Transl. Biophotonics.

[35] Galdran A., Costa P., Campilho A. Real-Time Informative Laryngoscopic Frame Classification with Pre-Trained Convolutional Neural Networks. Proceedings of the 2019 IEEE 16th International Symposium on Biomedical Imaging (ISBI 2019).

